# Detailed Analysis of Gene Polymorphisms Associated with Ischemic Stroke in South Asians

**DOI:** 10.1371/journal.pone.0057305

**Published:** 2013-03-07

**Authors:** Sunaina Yadav, Nazeeha Hasan, Thomas Marjot, Muhammad S. Khan, Kameshwar Prasad, Paul Bentley, Pankaj Sharma

**Affiliations:** 1 Imperial College Cerebrovascular Research Unit (ICCRU), Imperial College London, London, United Kingdom; 2 Department of Neurology, All India Institute of Medical Sciences, New Delhi, India; University of Cambridge, United Kingdom

## Abstract

The burden of stroke is disproportionately high in the South Asian subcontinent with South Asian ethnicity conferring a greater risk of ischemic stroke than European ancestry regardless of country inhabited. While genes associated with stroke in European populations have been investigated, they remain largely unknown in South Asians. We conducted a comprehensive meta-analysis of known genetic polymorphisms associated with South Asian ischemic stroke, and compared effect size of the MTHFR C677T-stroke association with effect sizes predicted from homocysteine-stroke association. Electronic databases were searched up to August 2012 for published case control studies investigating genetic polymorphisms associated with ischemic stroke in South Asians. Pooled odds ratios (OR) for each gene-disease association were calculated using a random-effects model. We identified 26 studies (approximately 2529 stroke cases and 2881 controls) interrogating 33 independent genetic polymorphisms in 22 genes. Ten studies described MTHFR C677T (108 with TT genotype and 2018 with CC genotype) -homocysteine relationship and six studies (735 stroke cases and 713 controls) described homocysteine-ischemic stroke relationship. Risk association ORs were calculated for ACE I/D (OR 5.00; 95% CI, 1.17–21.37; p = 0.03), PDE4D SNP 83 (OR 2.20; 95% CI 1.21–3.99; p = 0.01), PDE4D SNP 32 (OR 1.57; 95% CI 1.01–2.45, p = 0.045) and IL10 G1082A (OR 1.44; 95% CI, 1.09–1.91, p = 0.01). Significant association was observed between elevated plasma homocysteine levels and MTHFR/677 TT genotypes in healthy South Asians (Mean difference (ΔX) 5.18 µmol/L; 95% CI 2.03–8.34: p = 0.001). Our results demonstrate that the genetic etiology of ischemic stroke in South Asians is broadly similar to the risk conferred in Europeans, although the dataset is considerably smaller and warrants the same clinical considerations for risk profiling.

## Introduction

South Asia comprising of India, Pakistan, Sri Lanka and Bangladesh, forms 20% of the world's populous and shoulders much of the global death burden from cardiovascular disease [1] with India reporting 930,985 cases of stroke in 2004 leading to 639,455 deaths and loss of 6.4 million disability adjusted life years [2]. The past decade has seen prevalence rates of stroke rise in South Asia from between ∼200 [3]–[6] to 545 per 100,000 persons [7], while incidence studies demonstrate exponential increases in stroke incidence rates of >800% over the past 30 years (e.g. from 13 to 123 per 100 000 persons per year between 1969 and 1993) [8], [9]. Studies of South Asian diasporas in the West have also shown a heightened prevalence and incidence of stroke and coronary artery disease compared to Caucasians [10], [11]. This is compounded by the fact that South Asians are developing stroke at a relatively young age, despite lower rates of alcohol and tobacco use [12]. Although 90% of the population attributable risk (PAR) for stroke has been suggested to be attributed to ten modifiable risk factors [13], this does not account for the occurrence of stroke in young unexposed populations and also fails to explain the development of stroke in only some individuals within a population that is uniformly exposed to environmental risk factors. As with Europeans [14], [15], it is likely that the causality of stroke in South Asians involves the complex interaction between genetic and environmental risk factors.

We undertook a comprehensive meta-analysis of all known genetic associations with ischemic stroke in South Asians and compare it to published results in different ethnic groups. We further sought to establish whether homocysteine, the putative biochemical intermediary of the MTHFR gene is associated with quantitative levels of risk [16] in South Asians as similarly shown in Europeans [14], [16].

## Methods

### (1) SNP analysis

#### Data searching

We identified all published case-control studies evaluating any gene polymorphisms and ischemic stroke in South Asians residing in their native countries and diasporas around the world. Electronic searches were conducted using Medline, EMBASE and Google Scholar. All published manuscripts up until and including August 2012 as well as letters, previous meta-analyses and abstracts were included. The retrieved studies were examined thoroughly to assess their appropriateness for inclusion in our study. The references of all identified publications were manually reviewed for additional studies and the PUBMED *‘relevant articles’* option was utilized. The following index terms along with ‘*and*/*or*’ as a Boolean operator were used: “South Asia” “India” “Pakistan” “Sri Lanka” “Bangladesh” for ancestry and “stroke genetics” “gene polymorphism” “gene mutation” “stroke genes” for genetics and “stroke” “cerebrovascular disease” “ischemic stroke” “brain infarction” “brain ischemia” for clinical phenotype.

#### Study selection

Study inclusion criteria were: (1) studies in populations of South Asian descent defined as Indian, Pakistani, Sri Lankan or Bangladeshi; (2) case-control studies where ischemic stroke was analyzed as a dichotomous trait; (3) stroke was confirmed using brain imaging with sub-acute (within 10 days) CT or MRI, and; (4) genotype frequency for both cases and controls was reported. Studies were excluded if: (1) subjects were <18 years age; (2) the genotype frequency was not reported and could not be obtained from authors and (3) stroke other than ischemic.

#### Data extraction

Data extracted from each study included: first author, journal, year of publication, stroke sub-type, and number of cases and controls for each genotype and SNP. Baseline characteristics for cases and controls were documented including mean age, gender, ethnicity and geographical location.

#### Data analysis

For each genetic variant for which data were available from at least two studies, a meta-analysis was carried out. Data was analyzed using Review Manager *v*5.0 and Comprehensive Meta-Analysis (CMA) *v*2.0. To test for strength of association for each gene variant, a pooled odds ratio (OR) and 95% confidence interval (CI) was calculated using a random effects model [17]. The strength of genetic association (risk or protection) for each polymorphism was considered statistically significant if a p-value of <0.05 was obtained. For each analysis an I^2^ test for heterogeneity [18] was performed, with significance set at p<0.05. Funnel plots and Egger regression intercept p-value (two-tailed) were used to determine probability of publication bias [19]. Comparison of ORs of genes with significant associations was made between ethnic populations by observing whether 95% confidence intervals overlapped.

### (2) MTHFR C677T – homocysteine phenotype comparison

For MTHFR C677T gene variant which had an associated biomarker homocysteine, we performed a separate analysis that produced an estimate of expected risk based upon genotype–biomarker, and biomarker–stroke, association studies, using mendelian randomization [14], [16], [20].

#### Data searching

Electronic searches were conducted using Medline, EMBASE and Google Scholar and all published manuscripts up until and including August 2012 were considered. The following index terms along with ‘*and*/*or*’ as Boolean operators were used for MTHFR: (MTHFR OR *Methylenetetrahydrofolate reductase* ) AND (gene OR genetic OR genotype OR polymorphism OR mutation), in combination with (MTHFR OR *Methylenetetrahydrofolate reductase* ) AND (homocysteine OR activity OR level); and 2) (MTHFR OR *Methylenetetrahydrofolate reductase*) AND (activity OR level) in conjunction with (cerebrovascular disease OR brain infarction OR stroke OR cerebral ischemia) and (South Asia or India or Pakistan or Sri Lanka or Bangladesh).

#### Study selection

The literature was searched for two types of studies: (Study 1) case-control studies reporting dichotomous and continuous data for plasma homocysteine levels in South Asians with ischemic stroke, and; (Study 2) studies linking plasma homocysteine levels with the MTHFR 677 wild type (CC) and homozygous mutant (TT) genotype in healthy South Asians. Control populations with genotype-homocysteine data from case-control studies on psoriasis, coronary artery disease and glaucoma were also included.

#### Data extraction

Data extracted from each study included: first author, journal, year of publication, stroke sub-type, and total number of participants. Baseline characteristics for cases and controls were documented including mean age, gender, ethnicity and geographical location. In addition for Study 1, plasma homocysteine levels as means with standard deviations and median with ranges in ischemic stroke cases and controls, and; for Study 2, plasma homocysteine levels as means with standard deviations and median with ranges in MTHFR 677 wild type (CC) and homozygous mutant (TT) genotype in healthy South Asians.

#### Data analysis

The methodology for a literature based mendelian randomization for MTHFR and homocysteine is well described [14], [16].Where values of plasma homocysteine were reported as medians and ranges, the mean and standard deviation were estimated using established models dependent upon sample size [21]. For both types of studies, mean homocysteine difference between ischemic stroke cases vs. controls and MTHFR TT vs. CC genotypes were calculated using the continuous data type inverse variance method in Review Manager *v*5.0. A pooled mean difference (ΔX) with 95% confidence interval was calculated for MTHFR TT *vs* CC genotypes using a random effects model. Mean difference in homocysteine levels between ischemic stroke cases vs. controls were converted to ORs and 95% confidence interval using Comprehensive Meta-Analysis (CMA) *v*2.0. Assuming a log-linear relationship, this OR was scaled with the ΔX change in homocysteine levels conferred by the MTHFR TT genotype to give the expected OR [16].

For each meta-analysis an I^2^ test for heterogeneity was performed, with significance set at p<0.05. Funnel plots and Egger regression intercept p-value (two-tailed) were used to determine probability of publication bias.

## Results

### Meta-Analysis of Gene Variants Associated with Ischemic Stroke

**Figure 1:**
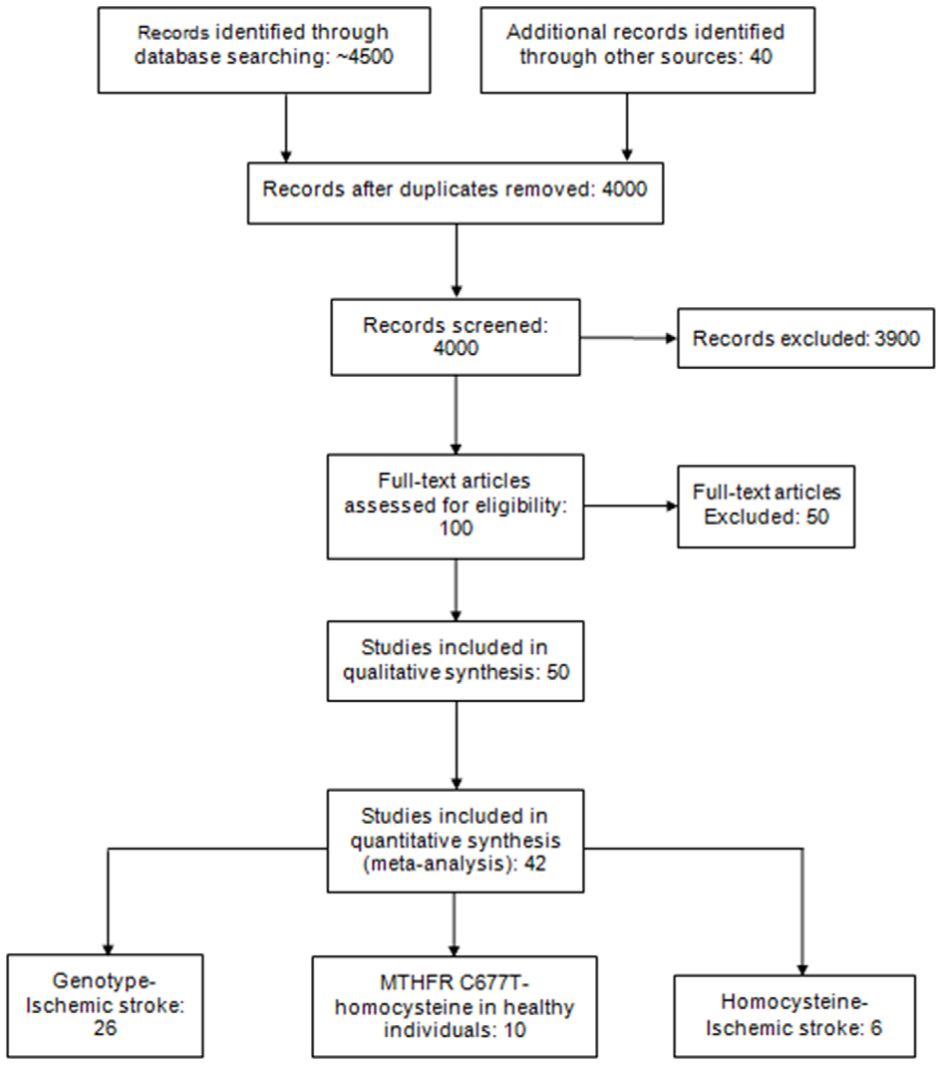
PRISMA statement flow diagram illustrating search strategy and studies included in the meta-analysis. *Articles excluded because they did not meet inclusion criteria and were missing genotype data.

Our search strategy identified ∼4500 potentially relevant studies of which 26 met the inclusion criteria allowing interrogation of 33 independent genetic polymorphisms in 22 different genes across 2529 stroke cases and 2881 controls (Figure 1 PRISMA flowchart). The majority of studies were from India (North and south) followed by Pakistan, Bangladesh, Malaysia and the United Kingdom (Table S1)

Relevant studies identified were; five for MTHFR C677T [22]–[26], three for PDE4D SNP 83 [27]–[29] and 2 studies each for eNOS 4a/4b [30], [31], ACE [32], [33], ApoE E4/E4 [34], [35], FVL G1691A [36], [37], PDE4D SNP 87 and 32 [38], [39] and IL10 [40], [41] genes. Two studies each reporting different stroke sub types (Cerebral Venous Thrombosis (CVT) and arterial paediatric ischemic stroke) were found for genes MTRR G66A [42], [43], MTR A2756G [42], [43], MTHFR A1298C [42], [43] and FVL A4070G [36], [42], [43], and therefore ORs for risk were not estimable. Prothrombin G20210A polymorphism was investigated in two studies [37], [44]. Homozygous or heterozygous variants were completely absent in all individuals studied and with only the wild type genotype being expressed an OR for risk was not estimable. One relevant study was identified for genes variants IL-1α (C889T) [45], CYP11B2 (C344T) [46], ESR1 (PVUII and XbaI) [47], α ADD1 (WG) [33], TNF α (G488A and G308A) [48], CYP4F2 (G1347A) [49], MDR-1 [50], t-PA (C7351T and I/D) [51], PAI-1 (4G/5G) [51], CBS (T833C) [52], Klotho (KL-VS and C1818T) [53], Factor XIIIB (V34L) [54], α1 antichymotrypsin (Ala15Thr) [55] and MMP3 (5A/6A) [48]. Of these IL-1α, CYP11B2, ESR1 (PVUII), α ADD1, TNF α (G488A), CYP4F2, MDR-1 and t-PA (I/D) were found to be significantly associated with ischemic stroke (Table S1).

### PDE4D SNP 83

The PDE4D SNP83 polymorphism was investigated in three studies covering Pakistan and North India [27]–[29] in a total of 1338 subjects (626 Ischemic stroke cases; 712 controls). A pooled OR of 2.20 (95% CI, 1.21–3.99, p = 0.01) was generated with a recessive random-effects model. There was no evidence of inter-study heterogeneity ([P_HET_] = 0.06, I^2^ = 65%).

### ACE I/D

The homozygous ACE D/D polymorphism was investigated in a total of 2 studies covering India [32], [33] in 693 subjects (355 ischemic stroke cases; 338 controls) providing a pooled OR of 5.00 (95% CI, 1.17–21.37, p = 0.03) with a recessive random-effects model. There was evidence of inter-study heterogeneity ([P_HET_] = 0.002, I^2^ = 90%).

### IL10 G1082A

The homozygous G1082A polymorphism was investigated in a total of 2 studies covering India in 1414 subjects (718 ischemic stroke cases; 696 controls) providing a pooled OR of 1.44 (95% CI, 1.09–1.91, p = 0.01) with a recessive random-effects model. There was no evidence of inter-study heterogeneity ([P_HET_] = 0.12, I^2^ = 59%).

### MTHFR/C677T

**Figure 2:**
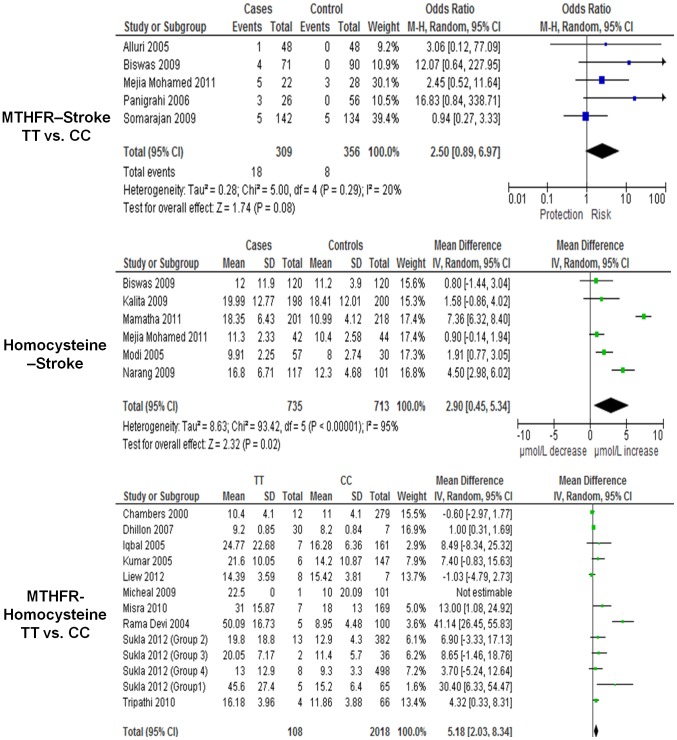
Meta-analysis, forest plot and pooled ORs of risk from studies investigating MTHFR C677T polymorphism (TT vs CC) -stroke, homocysteine-stroke and homocysteine-MTHFR C677T.

The homozygous MTHFR C677T polymorphism was investigated in seven studies [22]–[25], [36], [43], [56] out of which five studies covering North India and Malaysian Indians [22]–[25], [36] were included in the final analysis. These totalled 615 subjects (309 Ischemic stroke cases; 356 controls) providing a pooled OR of 2.50 (95% CI, 0.89–6.97, p = 0.08) with a recessive random-effects model (Fig. 2). There was no evidence of inter-study heterogeneity ([P_HET_] = 0.33, I^2^ = 14%).

### Biochemical Marker of Risk: MTHFR C677T and Homocysteine

Seven studies [22], [26], [57]–[61] that presented plasma homocysteine levels in South Asian ischemic stroke patients were subjected to a meta-analysis. One study [60] did not have normally distributed data with a threefold difference in mean homocysteine levels between cases and controls, and the study was excluded from the final analysis. The remaining six studies presented data as means and standard deviations (SD) as well as median with their lower and higher range. A mean difference in homocystiene between ischemic stroke cases and controls was found to be 2.90 µmol/L (p = 0.02, 95% CI 0.45–5.34) (Fig. 2) and this corresponded to an OR of 1.68 (95% CI 1.10–2.58), calculated using the CMA *v*2.0 software. We found 10 studies [62]–[71] linking homocysteine levels to the MTHFR 677 wildtype (CC) and homozygous mutant (TT) genotypes in healthy South Asians and calculated a mean difference (ΔX) of 5.18 µmol/L (p = 0.001, 95% CI 2.03–8.34) between the TT vs. CC genotypes in healthy individuals (Fig. 2). The expected odds ratio was calculated using the following formula:
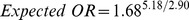
where 1.68 = OR associated with 2.90 µmol/L difference in homocysteine levels between stroke cases and controls

5.18 = 5.18 µmol/L mean difference in homocysteine levels between the MTHFR TT vs. CC genotypes in healthy individuals

2.90 = Mean difference of 2.90 µmol/L associated with an OR of 1.68

**Figure 3:**
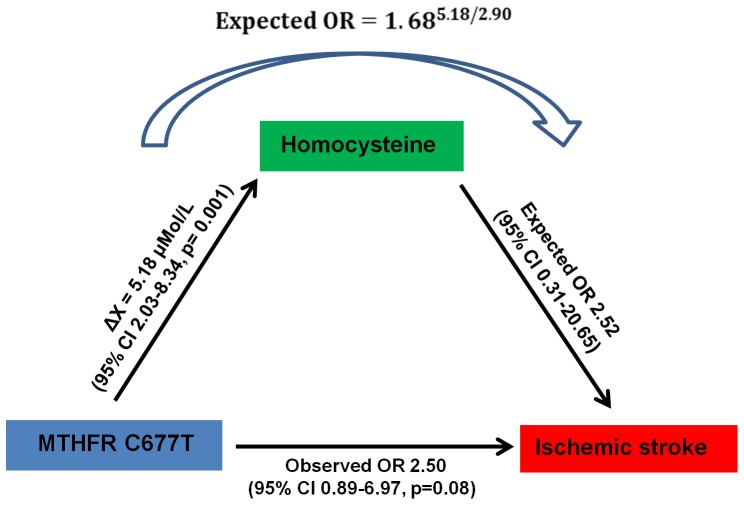
Mendelian Randomization to compare estimated risk with observed risk for gene polymorphisms associated with ischemic stroke. The mean difference (ΔX) was calculated from a meta-analysis relating MTHFR C677T genotype with homocysteine variation in healthy South Asians. The expected OR was calculated using the following formula: 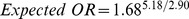 [16] where 1.68 was the OR associated with 2.90 µmol/L mean difference in homocysteine levels between stroke cases and controls.

The above calculation revealed an expected OR of 2.52. A 95% confidence interval for the logged odds ratio of 0.92 (LN 2.52) was obtained as 1.96 standard errors on either side of the point estimate as previously described by Bland et al [72]. Standard error (SE) was calculated as the square root of the sum of reciprocals of the frequencies i.e. number of cases and controls. The 95% confidence interval was calculated using the formula LN (OR) ±1.96×SE, which generated a range of −1.17 to 3.02. The antilog of these limits generated a 95% confidence interval for OR 2.52 as Exp (−1.17) = 0.31 to Exp (3.02) = 20.65. A meta-analysis relating MTHFR 677/TT vs. CC risk genotype to disease in our current study generated a pooled observed OR of 2.50 (95% CI, 0.89–6.97). The observed OR was close to the expected OR and its 95% confidence interval fell entirely within the confidence interval for the expected OR (Fig. 3).

## Discussion

We present, to the best of our knowledge, the most comprehensive genetic meta-analysis of ischemic stroke in South Asians from India, Pakistan, Sri Lanka and Bangladesh. Our findings suggest three genes (PDE4D, ACE I/D and IL10) to have statistically significant ORs for risk of ischemic stroke in a South Asian adult population and the totality of data supported MTHFR C677T as a further likely risk factor. Results from 12 other gene variants, although significant, were of insufficient power to allow robust conclusions. The remaining 17 gene polymorphisms (Table S1) failed to support any significant association either due to lack of sufficient studies, paucity of subjects studied and/or heterogeneity in the stroke sub-types investigated.

The two most significant risk associations, PDE4D SNP 83 and ACE I/D identified in our meta-analysis, were previously described as genetic risk factors for stroke in other ethnic groups [73]. We observed a doubling in the odds (OR 2.20; 95% CI, 1.21–3.99) or a ∼120% increase in the risk of developing stroke per copy of the risk allele of PDE4D SNP 83 for overall ischemic stroke. PDE4D gene encodes a phosphodiesterase enzyme that regulates cAMP levels in the body [74] and was first identified as a candidate gene for stroke by the DeCODE study [75]. However several attempts to replicate these findings failed [76], while some studies reported conflicting results [14], [77], [78]. These discrepancies have been attributed to possible problems in study design and analytical methods [79]. A recent meta-analysis by Yoon et al [80] identified SNP 83 as having a protective association with stroke in Asians (OR 0.79, 95% CI 0.69–0.90; p = 0.0005). The meta-analysis combined 4 studies of South East Asian origin and 2 studies of South Asian origin. While combining different ethnic populations for the analysis maybe a reasonable approach for an exploratory analysis of genetic risk factors, such assumptions may lead to fallacious results as studies have already proved that India has a unique genetic population sub-structure which cannot be imputed from other ethnic groups [81]. Other studies have also identified race-ethnic disparities for stroke risk factors such as hypertension and diabetes [82] as well as genes affecting stroke in different ethnic groups [83]. Our study included individuals of only South Asian ancestry from the Indian sub-continent and though the population size is small, it takes into account the need for independent analysis of South Asians.

ACE gene plays an important role in vascular physiology and structural integrity. Our study finds a high risk of ACE/DD variant in South Asians (OR 5.00: 95% CI, 1.17–21.37) which accounts for a five-fold increase in the risk of overall ischemic stroke per copy of the risk allele of the ACE DD variant relative to Europeans (OR 1.15: 95% CI, 1.06–1.25) [14]. Closer examination of this comparison revealed a very large confidence interval for our study largely due to low sample size (355 cases/338 controls vs. 4897 cases/13949 controls in Europeans) suggesting that the measured effect size is probably inflated. Partially overlapping confidence intervals further suggested that there may not be a statistical difference between the two groups. An alternate explanation could be the difference in the prevalent stroke sub type in South Asians as compared to other ethnic groups. The homozygous *D* allele is associated with hypertension in Asian Indians [84] and is also associated with preferential risk of small vessel disease (SVD) [85] which is the most common subtype of stroke found in South Asians [12], [86]. The polymorphism accounts for 47% of the total phenotypic variance of serum ACE [87] which is linked with quantitative levels of risk of disease [14] [47].

Interleukin 10 is an anti-inflammatory cytokine produced primarily by monocytes and type 2 T helper cells. We found IL10 to be associated with a 44% increase (OR 1.44; 95% CI 1.09–1.91) in the risk of overall ischemic stroke per copy of the risk allele of the IL 10 G1082A variant. IL10 is involved in various cellular processes such as inhibition of pro-inflammatory cytokines, suppression of antigen-presenting capacity of antigen presenting cells (APC) and stimulation of B cell maturation. IL10 forms part of an inflammatory genetic profile and elevated levels of IL10 post stroke have been implicated in severe neurological impairment and major adverse clinical outcomes [88], [89]. Since acute ischemic brain insult is known to trigger anti-inflamatories, mutations in IL10 may result in uninhibited effects of pro-inflammatory cytokines.

MTHFR C677T genotype is associated with hyperhomocystinemia among South Asians and Europeans [90] and has been shown to have a larger effect on homocysteine concentration in geographical regions of low folate consumption than in regions with high dietary folate intake [91]. Knowing whether there is an ethnic predisposition to hyperhomocystinemia is necessary as homocystiene levels can be effectively lowered by supplementing diet with folic acid, vitamin B12 and vitamin B6. This is important as the majority Hindu South Asian population has low plasma folate and Vitamin B12 levels [92], [93] which can be partly accounted for by their predominantly vegetarian diets or high temperature cooking methods which destroy folate. We sought to establish whether the putative biochemical intermediary, homocysteine, of the MTHFR C677T gene variant was associated with equivalent quantitative levels of risk [16] in South Asians. We compared effect sizes of gene-stroke associations with those predicted from independent biochemical data using a mendelian randomization strategy [14].

Although the genetic association between disease and gene for MTHFR C677T was weak (p = 0.08), the totality of data supported such an association and with its previously documented strong association in Europeans [94] led us to undertake this causal analysis. A mendelian randomization strategy for a causal association, was concordant with literature on Europeans [14], [16] with similar observed and expected odds ratios. Our study also showed a significant association (p = 0.02) between homocysteine levels and ischemic stroke where a 2.9 µMol/L elevation in plasma homocysteine levels increased the odds of stroke risk by 68%. This is in contrast to reports from European studies which have observed much lower odds of risk (24%) for a similar increment in plasma homocysteine levels [20]. Interestingly, the biochemical change C *vs* T also showed a high difference in homocysteine levels, ΔX value of 5.18 µMol/L, for South Asians, compared to Europeans ΔX of ∼2.3 µMol/L [14]. While it is possible this is an artifactual difference, another explanation is that healthy South Asians with CC genotype have overall higher levels of homocystiene than healthy CC genotype Caucasians resulting in a greater ΔX. Alternately, our study population maybe largely vegetarian and resultantly have high homocysteine levels [95]. Epidemiological studies [93] have already established ethnic differences in plasma homocysteine levels and these need to be followed up with comparative analysis of homocysteine levels in healthy South Asians and Europeans with MTHFR C677C genotypes.

As with all meta-analyses, a number of limitations to interpreting our data need to be reported. Possible publication bias [96] could be introduced by reporting only positive results published in the English language and unconscious exclusion of studies which may have reported contradictory results. Studies were smaller and much fewer in numbers leading to less robust conclusions compared to similar work in Europeans [97]. As an example, for ACE I/D polymorphism the availability of only 2 studies for the meta-analysis prevented us from conducting an iterative analysis to identify and remove the source of inter study heterogeneity ([P_HET_] = 0.002, I^2^ = 90%). The small sample size meant large a confidence interval and less statistical reliability of data. Lack of stroke TOAST classification amongst the selected studies also hampered sub-group analysis which is essential due to the heterogeneous etiology of ischemic stroke sub-types. Fewer studies also limited our ability to take the genetic stratification of the South Asian population [81] into account and we conducted a pooled analysis of both South and North Indian stroke cases.

Another major limitation was the inability to calculate the F statistic to determine the strength of the genetic instrumental variable (IV). A mendelian randomization strategy on individual patient level data involves the assessment of a causal effect of a phenotype on an outcome by using genetic IV [98].The magnitude of bias is determined by the F statistic for the strength of association between the IV and phenotypes and is essential to assess the appropriateness of the IV. In a literature based meta-analysis pooled F statistics can be calculated if each study included in the meta-analysis reports the study specific values. However none of the studies included in our meta-analysis reported the study specific F statistics.

In the current climate of GWAS studies and whole genome sequencing, the literature based meta-analysis has its own unique utility. Where large GWAS studies have failed to identify risk associations with gene variants such as *MTHFR* and *ACE*, literature based meta-analysis of comparable (or greater) power and sample size have been able to identify these genetic risk variants to be associated with stroke [14], [15]. Although the stand alone validity of individual candidate gene based studies remains inconclusive, a meta-analysis may reveal a true association. Our meta-analysis included studies that examined candidate genes previously identified in other vascular disorders such as MI in Europeans and South East Asians. Though this is a reasonable exploratory approach to understanding the genetics of stroke in South Asians, there is a clear need for larger GWAS strategies to truly understand the genetic underpinnings of stroke. A good starting point could be replication in an independent South Asian stroke population of gene variants identified in large statistically powered GWAS studies conducted in Europeans. The Wellcome Trust Case Control Consortium 2 (WTCCC2) and the International Stroke Genetics Consortium (ISGC) have successfully identified novel genes associated with stroke risk (PITX2, ZFHX3, 9p21 locus and HDAC9) [99] while other GWAS studies have highlighted the subtype specific nature of these genetic effects [100]. The recent METASTROKE [100], [101] meta-analysis which included 15 stroke cohorts comprising of 12,000 cases and 60,000 controls validated these findings but failed to identify any new genetic risk variants. Replication of SNPs from related cardiovascular GWAS studies found one novel association with gene *PHACTR1* which suggests that detection of any new genetic risk variants will rest on proper stroke subtyping. Though the effect sizes for these genes are small, it is likely that they are true associations as compared to genes identified in smaller underpowered candidate gene based studies.

Our work supports a genetic etiology of ischemic stroke in South Asians but the dataset is considerably smaller compared to those of European descent. We show no major differences in risk associations for previously studied stroke susceptibility genes between South Asians, Europeans and South East Asians. However it would be fallacious to assume a literal comparison between studies that are not statistically at power and hence there is a clear need for large prospective well powered GWAS studies in South Asians, as has been done in Europeans.

## Supporting Information

Table S1 **Summary table of gene polymorphisms associated with risk of ischemic stroke in South Asians.**
(DOCX)
