# Supplementary material for: Detailed Analysis of Gene Polymorphisms Associated with Ischemic Stroke in South Asians
Source: PLoS One. 2013 Mar 7;8(3):e57305. doi: 10.1371/journal.pone.0057305 (PMC3591429; doi:10.1371/journal.pone.0057305)
Supplement: Table S1 — Summary table of gene polymorphisms associated with risk of ischemic stroke in South Asians. (DOCX) [file pone.0057305.s001.docx]

**ONLINE SUPPLEMENTARY MATERIAL**

**Detailed analysis of Gene Polymorphisms Associated with Ischemic Stroke in South Asians**

**Yadav et al**

**Supplementary Table**

**Table S1:** Summary table of gene polymorphisms associated with risk of ischemic stroke in South Asians. All comparisons are based on a recessive genetic model i.e. homozygous mutant alleles (MM) vs. total alleles (W+) where W and M denote wild type and mutant alleles respectively and + can denote either W or M.

| **Gene** | **Polymorphism**  **(Risk Allele)** | **Model** | **Study** | **Cases** | | | **Control** | | | **Pooled OR**  **(95% CI)** | **p** |
| --- | --- | --- | --- | --- | --- | --- | --- | --- | --- | --- | --- |
|  |  |  |  | **N** | **Age (years)** | **Gender (F/M)** | **N** | **Age (years)** | **Gender (F/M)** |  |  |
| MTHFR | C677T (T) | Recessive | Biswas 2009  (J of Stroke and CVD) ∞ | 71 | <40 | NR | 90 | <40 | NR | 2.50 (0.89-6.97) | 0.08 |
|  |  |  | Alluri 2005∞ | 48 | 7-78 | 9/60 | 48 | NR | NR |  |  |
|  |  |  | Somarajan 2011∞ | 142 | 54±15.9 | 55/152 | 134 | 55.25±10.65 | 62/126 |  |  |
|  |  |  | Panigrahi 2006∞ | 26 | 12 (1-42) | 10/32 | 56 | NR | NR |  |  |
|  |  |  | Mejia Mohamed 2011⌂ | 22 | 61.0±10.1 | 58/92 | 28 | 60.6±7.1 | 71/71 |  |  |
| ACE | I/D (D) | Recessive | Kalita 2011 (Clin. Chim. Acta) ∞ | 193 | 56.5±2.8 | 52/141 | 188 | 55.7±12 (F)  54.3±9.7 (M) | 69/129 | 5.00 (1.17-21.37) | 0.03 |
|  |  |  | Munshi 2008 (J of Neur Sc.) ◊ | 162 | 52.83±4.04 | 42/120 | 150 | 54.76±1.04 | 39/111 |  |  |
| ApoE | E4/E4 | Recessive | Luthra 2002 (Clin Gen.) | 63 | 56.4±13.1 | NR | 57 | 39.4±8.0 | NR | 2.15 (0.75-6.19) | 0.16 |
|  |  |  | Chowdhury 2001 (J of Epid.)◦ | 147 | 57.9±11.1 | 30/117 | 190 | 60.3±9.6 | 61/129 |  |  |
| ENOS | 4a/4b (aa) | Recessive | Majumdar 2010 (JAT) ◊ | 175 | 29.71±10.82 | 54/123 | 214 | 28.42±7.87 | 72/147 | 1.87 (0.77-4.59) | 0.17 |
|  |  |  | Munshi (BRB 2010) ◊ | 357 | 48.01±15.25 | 93/264 | 283 | 47.05±16.75 | 73/210 |  |  |
|  | T786C (C) | Recessive | Majumdar 2010 (JAT) ◊ | 129 | 29.71±10.82 | 54/123 | 129 | 28.42±7.87 | 72/147 | 1.93(0.63-5.93) | 0.25 |
|  | G894T (T) | Recessive | Majumdar 2010 (JAT) ◊ | 172 | 29.71±10.82 | 54/123 | 214 | 28.42±7.87 | 72/147 | 0.64 (0.18-2.27) | 0.49 |
| PDE4D | SNP 83 | Recessive | Munshi 2009 (JNS) ◊ | 250 | 48.53±16.34 | 62/188 | 250 | 47.01±17.78 | 65/185 | 2.20 (1.21-3.99) | 0.001 |
|  |  |  | Saleheen 2005 (Stroke) ▫ | 200 | 62.4±12.4 | 82/118 | 250 | 54.1±8.87 | 87/163 |  |  |
|  |  |  | Banerjee 2008 (Brain Res Bul.) ◊ | 176 | 58.6±14.2 | 63/113 | 212 | 57.4±8.8 | 69/143 |  |  |
|  | SNP 87 | Recessive | Munshi 2009 (JNS) ◊ | 250 | 48.53±16.34 | 62/188 | 250 | 47.01±17.78 | 65/185 | 1.17 (0.71-1.94) | 0.54 |
|  |  |  | Saleheen 2005 (Stroke) ▫ | 200 | 62.4±12.4 | 82/118 | 250 | 54.1±8.87 | 87/163 |  |  |
|  | SNP 32 | Recessive | Munshi 2009 (JNS) ◊ | 250 | 48.53±16.34 | 62/188 | 250 | 47.01±17.78 | 65/185 | 1.57 (1.01-2.45) | 0.045 |
|  |  |  | Saleheen 2005 (Stroke) ▫ | 200 | 62.4±12.4 | 82/118 | 250 | 54.1±8.87 | 87/163 |  |  |
|  | SNP 41  (T) | Recessive | Munshi  (Gene 2012) ◊ | 516 | 49.3± 17.34 | 156/360 | 513 | 49.01±16.78 | 16.78 | 1.76 (1.25-2.48) | 0.001 |
|  | SNP 56  (T) | Recessive | Munshi  (Gene 2012) ◊ | 516 | 49.3± 17.34 | 156/360 | 513 | 49.01±16.78 | 16.78 | 3.25 (2.30-4.59) | <0.00001 |
|  | SNP 59736747 T>G (G) | Recessive | Munshi  (Gene 2012) ◊ | 516 | 49.3± 17.34 | 156/360 | 513 | 49.01±16.78 | 16.78 | 1.23 (0.50-2.99) | 0.66 |
| IL 10 | G1082A | Recessive | Munshi 2010  (Cytokine) ◊ | 480 | 49.3±17.34 | 123/357 | 470 | 47.01±16.78 | 155/351 | 1.44 (1.09-1.91) | 0.01 |
|  |  |  | Sultana 2011 ◊ | 238 | 53.72±11.11 | 74/164 | 226 | 54.06±10.98 | 105/121 |  |  |
| PAI-1 | 4G/5G | Recessive | Babu 2012 (Gene) ◊ | 516 | 49.3 | 156/360 | 513 | 49.01 | 155/358 | 1.33 (0.83-2.15) | 0.24 |
| Cystathionine Beta-synthase | T833C/844ins68  (C)* | Recessive | Chandra 2006  (Neur. India) | 30 | NR | NR | 138 | NR | NR | 0.40 (0.049-3.2) | 0.39 |
| IL-1 α | C889T  (T) | Recessive | Banerjee (BRB 2008) ∞ | 176 | 58.6±14.2 | 63/113 | 212 | 57.4±8.8 | 69/143 | 0.53 (0.33-0.86) | 0.009 |
| Factor XIIIB | V34L | Recessive | Kain 2005 | 143 | 63±34 | 40/40 | 146 | 61±35 | 42/38 | 3.16 (0.32-31.08) | 0.32 |
| α1 antichymotrypsin | Ala15Thr | Recessive | Somarajan 2009∞ | 272 | 53±16.7 | 75/197 | 188 | 54.8±10 | 65/ | 0.68 (0.40-1.17) | 0.17 |
| ADD1 | G/W460 | Recessive | Kalita (CCA 2011) | 193 | 56.5 (2-83) | 52/141 | 188 | 54.3±9.7 (M) 55.7±12 (F) | 69/129 | 1.38 (0.43-4.42) | 0.59 |
| CYP11B2 | C344T | Recessive | Munshi  (JNS 2010) ◊ | 403 | 49.3±17.34 | 116/287 | 394 | 47.01±16.78 | 115/279 | 0.43 (0.29-0.63) | 0.0001 |
| ESR | Pvu II | Recessive | Munshi  (CCA 2011) ◊ | 400 | 49.3±17.34 | 115/285 | 380 | 47.01±16.78 | 102/278 | 1.92 (1.24-2.99) | 0.004 |
|  | Xba I | Recessive | Munshi  (CCA 2011) ◊ | 400 | 49.3±17.34 | 115/285 | 380 | 47.01±16.78 | 102/278 | 1.59 (0.92-2.75) | 0.09 |
| Klotho | KL-VS | Recessive | Majumdar 2010 ◊ | 460 | 41.76±16.34 | 258/202 | 574 | 40.36±11.42 | 321/253 | 1.26 (0.56-2.84) | 0.57 |
|  | C1818T | Recessive | Majumdar 2010 ◊ | 460 | 41.76±16.34 | 258/202 | 574 | 40.36±11.42 | 321/253 | 0.81 (0.48-1.38) | 0.44 |
| α-ADD1 | WG | Recessive | Kalita (CCA 2011)∞ | 193 | 56.5±2-83 | 52/141 | 188 | 55.7±12 (F)  54.3±9.7 (M) | 69/129 | 1.37 (0.43-4.39) | 0.60 |
| TNF α | G308A | Recessive | Sultana 2011 ◊ | 238 | 53.72±11.11 | 74/164 | 226 | 54.06±10.98 | 105/121 | 1.39 (0.52-3.72) | 0.5 |
|  | G488A | Recessive | Munshi (EJN 2011) ◊ | 525 | 49.3 | NR | 500 | 47.01 | NR | 1.91 (1.32-2.76) | 0.0006 |
| MMP-3 | 5A/6A | Recessive | Munshi (EJN 2011) ◊ | 525 | 49.3 | NR | 500 | 47.01 | NR | 0.87 (0.58-1.29) | 0.9 |
| CYP4F2 | G1347A | Recessive | Munshi  (M B R 2012) ◊ | 507 | 49.3±17.34 | 144/363 | 487 | 49.01±16.78 | 131/356 | 1.58 (1.16-2.15) | 0.004 |
| MDR 1 | C3435T | Recessive | Sharma (Neur. Sciences 2011) ◊ | 560 | 49.3±17.34 | 167/393 | 560 | 49.01±16.78 | 173/387 | 2.66 (1.98-3.55) | <0.000001 |
| LPL | Hind III | Recessive | Munshi (JNS 2012) ◊ | 525 | 49.3±17.34 | 151/374 | 500 | 49.01±16.78 | 143/357 | 0.65 (0.43-0.97) | 0.03 |
| tPA | C7351T | Recessive | Babu (Gene 2012) ◊ | 516 | 49.3±17.34 | 156/360 | 513 | 49.01±16.78 | 155/358 | 1.14 (0.74-1.77) | 0.56 |
|  | I/D | Recessive | Babu (Gene 2012) ◊ | 516 | 49.3±17.34 | 156/360 | 513 | 49.01±16.78 | 155/358 | 1.74 (1.16-2.62) | 0.008 |

∞ Studies from North India, ⌂Studies from Malaysia, ◊ Studies from South India, ◦ Studies from Bangladesh, ▫ Studies from Pakistan
